# Elite Badminton Is Getting Older: Ages of the Top 100 Ranked Badminton Players from 1994 to 2020

**DOI:** 10.3390/ijerph182211779

**Published:** 2021-11-10

**Authors:** Pablo Abián, Luis Simón-Chico, Alfredo Bravo-Sánchez, Javier Abián-Vicén

**Affiliations:** 1 Faculty of Humanities and Social Sciences, Comillas Pontifical University, 28049 Madrid, Spain; pabloo9@hotmail.com; 2 Performance and Sport Rehabilitation Laboratory, Faculty of Sport Sciences, University of Castilla-La Mancha, 45071 Toledo, Spain; luigiss98@gmail.com (L.S.-C.); alfredo.bravo@uclm.es (A.B.-S.)

**Keywords:** badminton, gender, ageing, sport and performance

## Abstract

The purpose of this study was to determine the evolution of the age of badminton players in the top 100 of the World Ranking for men and women from 1994 to 2020. Data were collected from badminton players participating in the top 100 World Rankings (4800 entries: 1233 players; 595 men and 638 women) from 1994 to 2020. The mean age of the top 100 and the average highest ranking of the players were analysed for both genders. The mean age of the male players in the World Ranking increased from 23.7 ± 3.2 years in 1994 to 26.3 ± 4.4 years in 2020 (*p* < 0.001) and in female players, from 22.8 ± 3.8 years in 1994 to 24.7 ± 3.3 years in 2020 (*p* < 0.001). In addition, women recorded a younger age at entry into the top 100 and when reaching their best ranking. Additionally, there has been a clear increase in Asian players in the top 100 of the World Ranking in recent years, reaching over 60%. These data could be used to develop and organise training plans in this sport, optimising and maximising players’ performance.

## 1. Introduction

Age has been determined to be one of the main factors influencing sports performance [1], with physical exercise and training being influential aspects in the delay of the muscular–tendinous deterioration caused by ageing [2]. The last few decades have seen an increase in age regarding high-level athletes [3,4,5,6]. Moreover, Schulz and Curnow [7] asserted that the nature of the sport can affect the moment of the highest performers, being earlier in sports that rely heavily on strength, speed and power, and later in sports in which endurance and cognitive abilities have a more prominent role, and which were led by athletes of around 30 years old.

Technical ability, tactical knowledge and the physical condition of the athletes are essential elements linked to sports success, and are influenced by athletes’ ages and training loads [8]. Sports planning and talent picking can be determined by the age at which the highest performance is reached [6]. Longo et al. [6], by analysing the age of maximum performance in 40 disciplines at the 2012 Olympics, noted that 72% of the athletes were in the 20–30 age range, and only 1% passed the 40-year-old barrier, although the results varied widely depending on the discipline. Schulz and Curnow [7] showed that the age at which peak performance was achieved varied widely between sports disciplines, but in general, men reached their peak performance later than women. There was also a tendency for those sports that require specific technical abilities to have a higher number of younger players, whereas sports requiring tactical abilities were played by older athletes [7].

Economic and social changes such as the higher income of athletes and women’s increasing presence in sports competitions are some of the decisive factors for understanding the rise in the age of maximum performance [9]. In this regard, Gallmann et al. [3], investigating the influence of age in elite triathletes from the World Championships known as “Ironman Hawaii”, showed that the age of the first classified in the tournament from 1983 to 2012 had risen (from 27 ± 2 to 34 ± 3 years in men and from 26 ± 5 to 35 ± 5 years in women) and the race time had progressively improved. Another study based on tennis players showed that women enter the top-100 of the World Ranking (WR) and also reach their best position sooner, but remain in the top 100 less time than men [4].

Several studies have found that the muscle injury incidence is more frequent in older badminton players [10,11], but to date, research on high-performance capabilities regarding badminton players is scarce, and no studies have been conducted on the influence of age and the performance of the players. The main purpose of this study was to determine the evolution of the age of badminton players in the top 100 of the WR for men and women from 1994 to 2020. Secondary purposes were established to determine the age of badminton players when recording their best ranking, to establish the number of years that they remain in the top 100 of the WR, to determine the percentage of players from each continent and to analyse the differences between sexes. We hypothesised that badminton players would currently be older than they were a few years ago, they would reach their best ranking later and would be remain in the elite for more time; moreover, the women would be younger and reach their best ranking early than men.

## 2. Materials and Methods

### 2.1. Participants and General Procedure

Data were collected through the World Badminton Federation web site [12], spanning 26 years of the WR, from the first list in 1994 until the last list in March of 2020 (the data for the years 2006 and 2007 could not be obtained so were not included in the analysis). Data were collected on the top 100 badminton players of the WR: 4800 entries and 1233 players were recorded, 595 men and 638 women.

Each year’s December data on the WR (excluding March in 2020 due to the COVID-19 pandemic) were used to carry out this research. In the WS modality, surname changes after marriage were investigated in order to perform a proper follow-up of each player’s personal itinerary and avoid duplicated profiles.

The players’ best ranking was established according to the highest WR position obtained during their sports career. For players who reached the highest positions in several years (such as number 1), the procedure was to use the youngest age at which they reached that position. Data from the first year (1994) were excluded for the best ranking analysis [4].

The investigation was supervised by a Research Ethics Committee according to the Declaration of Helsinki. The Committee declared that this research required no approval.

### 2.2. Variables

The analysed calendar years (from 1994 to 2020) and the modality (men’s and women’s singles) were established as grouping variables.

Dependent variables were: mean age of the top 100 players, mean age at the first entry into the top 100, mean age of best ranking, frequency related to age in the top 100, frequency distribution according to age in the top 100 and number of years remaining in the top 100.

### 2.3. Statistical Analysis

The following computer programmes were used: Microsoft Excel spreadsheet (Microsoft, Spain) to store the data and the SPSS v. 26.0 Software (SPSS Inc., Chicago, IL, USA) to carry out the statistical analyses. Descriptive, normality and inferential statistical tests were also used. Data from the current research are presented as the mean ± standard deviation or as frequencies. A two-way ANOVA was used to calculate the difference between group rankings (top 100, top 75, top 50, etc.) and sex (group × sex), and a two-way ANOVA of repeated measures to compute the differences of ages between years and sex (time × group). After the F significance tests, the Bonferroni settings were used for pairwise comparisons. Chi-squared analysis was used to compare the frequency distribution. Effect size (ES) statistics were used to quantify the magnitude of the difference in pairwise comparisons, according to the formula proposed by Cohen [13]. Statistical significance was set at *p* < 0.05.

## 3. Results

**Figure 1:**
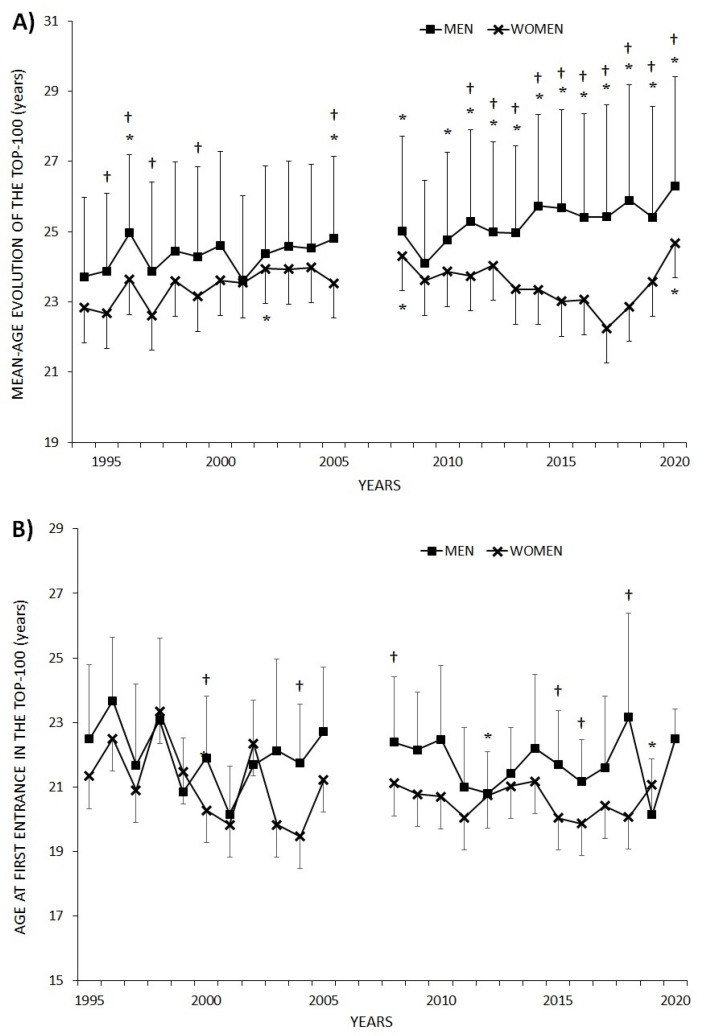
Evolution of average age in badminton players (both men and women) present in the World Ranking top 100 from 1994 to 2020 (**A**). Evolution of the average age of badminton players’ on their first entry in the World Ranking top 100 from 1995 to 2020 (**B**). * Significant differences in regard to the first record of the series in 1994 (*p* < 0.05); ^†^ Differences between men and women in paired data (*p* < 0.05).

The evolution of mean age in the 100 best badminton players from the WR in the last 26 years (1994 to 2020) is shown in Figure 1A. A progressive increase was observed in men’s average age, which became significant from 2010 onwards, in comparison to 1994 (1994 = 23.7 ± 3.2 years vs. 2020 = 26.3 ± 4.4 years; *p* < 0.001; ES = 0.7). Regarding women’s results, this increase in age was only significant in 2002, 2008 and 2020 (compared to 1994, *p* < 0.05), showing a variation from 22.8 ± 3.8 years in 1994 to 24.7 ± 3.3 years in 2020 (*p* < 0.001; ES = 0.5). It is evident that men’s top 100 average age was higher than women’s, particularly (*p* < 0.05) from 2010 to 2020, showing the greatest difference in 2017.

Figure 1B shows the evolution of the mean age at the first entry into the WR top 100 of badminton players in the last 25 years (1995 to 2020). Regarding women, no significant differences were found in any year in comparison to the first recorded year (1995, *p* > 0.05), whereas men’s data show a lower age concerning the first entry into the top 100 in 2001, 2012 and 2019 (compared to 1995, *p* < 0.05). Men’s average age at the first entry into the top 100 was higher than women’s, and significantly higher (*p* < 0.05) in 2000, 2004, 2008, 2015, 2016 and 2018, with the greatest difference in 2018.

**Figure 2:**
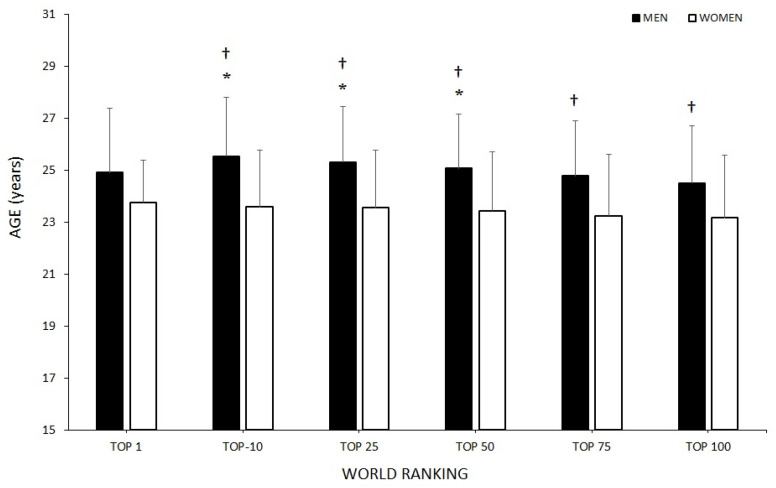
Average age of badminton players in reaching their peak performance considering their best career ranking in the World Ranking. * Significant differences in regard to the top 100 (*p* < 0.05); ^†^ significant differences in regard to women players (*p* < 0.05).

Mean age related to the best ranking in badminton players is shown in Figure 2. The best ranking in their sports career was significantly higher in men compared to women in the top 10, top 25, top 50, top 75 and top 100, and women reached their best ranking at a younger age than men (men = 25.6 ± 3.2 years vs. women = 23.6 ± 3.1 years; *p* < 0.05; ES = 0.6).

**Figure 3:**
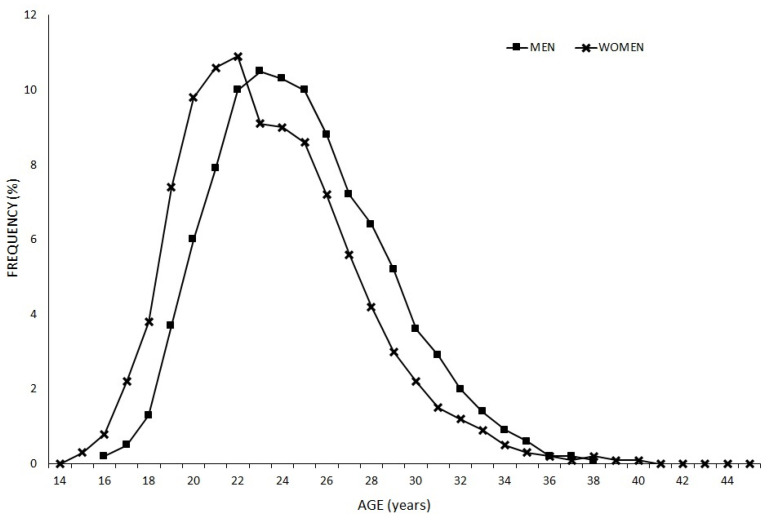
Frequency distribution in relation to age in badminton players (both men and women) with 1-year intervals in the analysed period (1994–2020).

Frequency in relation to WR top 100 players’ ages at one-year intervals during the analysed period (1994–2020) is shown in Figure 3. Both the men’s and women’s curves show an inverted U-shaped distribution, with the men’s range being between 21 and 28 years old and the women’s between 19 and 26 years old. The maximum frequency of men was in the 23–24-year-old group, and of women it was in the 21–22-year-old group.

**Figure 4:**
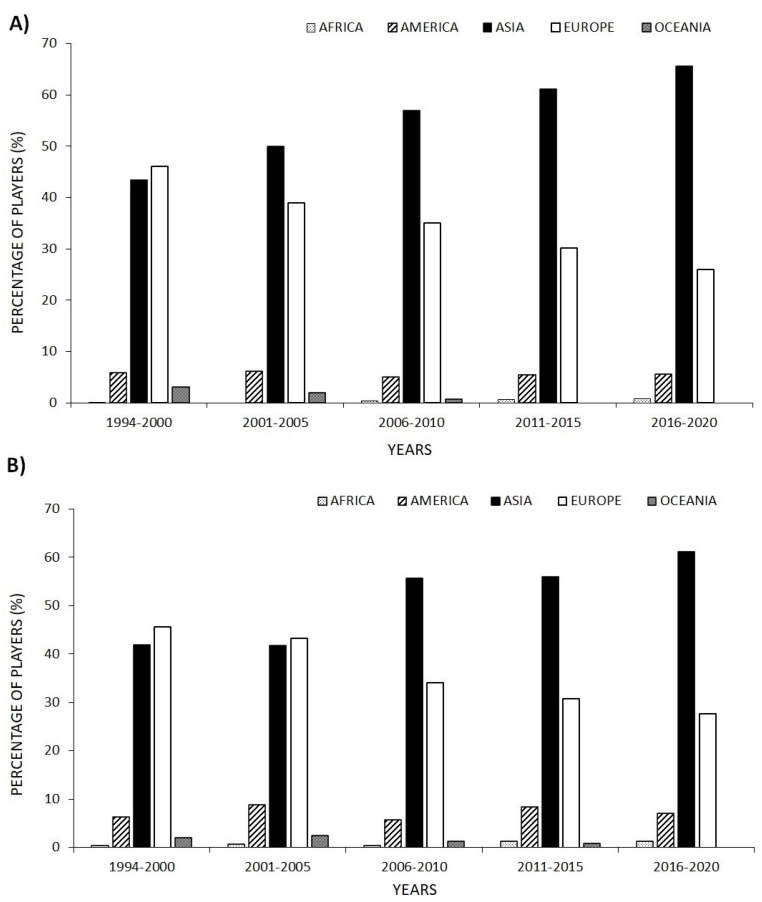
Percentage of players from each continent in the World Ranking top 100 (both men (**A**) and women (**B**)).

Regarding the number of players per continent in the WR top 100, both men and women from Asia and Europe showed a similar percentage in the first years (1994–2000) with ~46% and ~43%, respectively. However, from 2006 onwards, the presence of Asian players became more significant, surpassing 50%, followed by European players with ~30% and with fewer than 10% American players. Africa and Oceania were almost totally unrepresented, and even absent in some years. More than 60% of the players were Asian in the 2016–2020 period. (Figure 4).

The frequency distribution according to players’ ages has varied in some age ranges during the last few decades (Table 1). In the 1990s, most badminton players (men and women) were in the 21–25-year-old range (men: 51.6%; women: 50.6%), with lower frequencies for older players (>30 year). In relation to men’s results and despite the decrease in relationship to the 2000s, the 21–25-year-old range still represented the highest number of players (45.4%). However, the range of the over-30 years age group has tripled in regard to the 1990s (1990s: 3.9% vs. 2010s: 11.9%). Comparing women and men in the 2010s, there was a higher percentage of women players in the 14–20-year-old range and a greater percentage of men in the 26–30 years and 30+ years ranges.

Regarding the badminton players’ continuity in the WR top 100, men remained an average of 4.2 ± 3.4 years, whereas women remained 3.9 ± 3.1 years. More than 55% of the players (men and women) who reached the top 100 remained there for 1–3 years, and only 8.9% of men and 7.2% of women stayed for 10 years or more. (Table 2).

## 4. Discussion

The aim of this study was to establish the evolution in regard to age in badminton players (men and women) from the WR top 100 from 1994 to 2020, finding out the age at which they recorded their best ranking as well as discovering the number of years remaining in the top 100 and describing possible differences between sexes. The main results of this research were the following: (a) mean-age of the WR top 100 has increased in both men and women in the last few years being higher in men than women from 2011 on; (b) men’s ages at first entry in the WR top 100 decreased in 2001, 2012 and 2019 in comparison with 1995; (c) continuity in the WR top 100 was less than 6 years for ~75% of the players, both men and women; (d) men’s ages of best ranking was ~25 years, whereas women’s was ~23 years, with women reaching their best ranking for the top 10, top 25, top 50, top 75 and top 100 at a younger age; (e) age frequency distribution regarding the analysed period (1994–2020) shifts to earlier ages in women (21–22 years) in comparison to men (23–24 years); (f) from 2006, Asian players have represented more than 60% of the top 100, followed by European players (~30%) and American players with less than 10%.

The evolution of the mean age of the badminton players showed an ageing of the elite men players from 2010, and in the last year, 2020, for the women players. This increase in the mean age of the best athletes has already been described in relation to other disciplines such as triathlon [3], swimming [14,15] and tennis [4] in both men and women. In the same vein, Gallo-Salazar et al. [4] reported a significant increase in relation to age among the best tennis players from 2010, which may be due to improved training programmes, greater knowledge about the sport and better injury prevention [16,17,18]. Furthermore, thanks to coaches’ training and the use of new technologies, tactical aspects have been gaining more relevance in badminton [19], which may be an explaining factor for the increase in the average age of elite players [6], lengthening the badminton player’s career.

Improvements in the quality of life are linked to increases in the population’s average age [20], which may also have had a positive effect on the longer sports careers of badminton players. The increases were 2.9 and 1.8 years for men and women, respectively, in relation to the age recorded in the first year analysed in this study; values that are similar to those of other racket sports such as tennis [4] and higher when compared to other team sports such as football [5]. In addition, other factors, such as badminton’s lower injury ratio [21] in comparison to contact sports [22], may affect this increase in the average age of elite players.

The evolution of age in men and women badminton players from the top 100 results show that the years following Olympic Games (1996, 2000, 2004, 2008, 2012 and 2016) tended to present lower average ages in both modalities. This phenomenon may be due to the symbolic weight of the Olympics for any sports player, who may prolong their career aiming to take part in such a key event. Whether they succeed or not, it is common for players who reached their peak performance several years before to withdraw from elite competition or even retire.

Badminton players in the men’s singles also entered the top 100 for the first time at an older mean age (~22 years) than women (~21 years); similar results were previously obtained by Gallo-Salazar et al. [4] in tennis. Similarly, women reach their best ranking position earlier within all analysed bands: top 10, top 25, top 50, top 75 and top 100 of the WR. This may be because maturation and the psychomotor development of women are earlier than for men [23,24,25]. Our data showed a decrease in the percentage of players of aged 20 years or less and an increase in the percentage of players aged over 30 years during the last decade, similar to results recorded in tennis [4]. Thus, this higher percentage of male players aged 30 years or older may hinder men reaching better rankings at earlier ages, reflecting the gender gap in sports, as presented in Thibault et al. [26].

Regarding the number of players from each continent in the WR regarding both men and women, data show a significant increase in the presence of Asian players, who have represented around 60% of the top 100 from 2006 onwards. This phenomenon could be explained by the increasing number of badminton players in the world, as noted by Phomsoupha and Laffaye [27], but more specifically, because of the rise in the number of professional badminton players in Asia. Badminton is currently the most popular sport in such countries as China, Indonesia, Malaysia and Korea [28,29].

This research is subject to some limitations that will be analysed to understand the applicability of its results. To begin with, data from 2006 and 2007 could not be gathered; thus, these years were excluded from the analysis. Secondly, the game system was modified in 2006, which may have had an influence on maximum performance and players’ average age, as well as physiologically, due to the adaptation to the new scoring format [19,27,30]. Thirdly, this research assumed that the maximum performance of a player is related directly to their best position reached in the WR at the end of every calendar year. However, peak performance may be reached at any point of the year or at any specific tournament, which may not be reflected in the WR ranking at the end of the year. Lastly, as in any sport, badminton performance can be influenced by several factors such as injuries, qualification and access to different tournaments, group-stage draws, as well as leaderboards or personal issues from each player, which have not been considered in this research.

### Practical Applications

This research aims to shed some light on the possible links between age, sex and badminton performance. A precise description of maximum performance ages in men and women can be important information for boosting the strategies related to sports career planning. Using the data from this research as a starting point, badminton players and coaches can estimate the age of achieving their peak performance and how many years they can remain in elite competition. Considering the increase in age from players from the top 100, many others can be encouraged to continue trying despite their age. Data from this study may be of use for federations and coaches as well as for training programmes focused on long-term career planning.

## 5. Conclusions

In conclusion, this research shows that professional badminton is ageing, both in men’s and women’s singles, because the age of the 100 best players from the WR has increased from 23.7 ± 3.2 years in 1994 to 26.3 ± 4.4 years in 2020 for men, and from 22.8 ± 3.8 years in 1994 to 24.7 ± 3.3 years in 2020 for women. Moreover, women enter the top 100 of the WR earlier than men, showing the greatest difference in 2018, and reaching their best ranking at a younger age than men.

However, the increase in the average age of the players is not due to later entry into the top 100. The entry of women into the top 100 has remained stable, whereas in the men’s game, it has even been brought forward in some years. This increase in the average age of the players is the result of the professional sporting life of badminton players becoming longer. Badminton is becoming more Oriental; from 2006 onwards, more than 60% of the WR top 100 have been Asian players, because for many countries on the Asian continent, this sport is part of their culture and is therefore becoming the most important sport.

This information can be very useful for players, coaches, clubs and federations at both international and national levels, because the results obtained in this research show that the age of the athlete influences their performance. Taking this into account, coaches will be able to develop different performance and training plans with short- or long-term goals depending on the age of their athletes.

## Figures and Tables

**Table 1:** Frequency distribution from the World Ranking top 100 (both men and women) according to age in the last few decades (1990s, 2000s and 2010s).

		Decades		
Sex	Age Bracket	1990s	2000s	2010s
MEN	14–20 years (%)	14.5 *^†^	12.1 ^†^	10.2 ^†^
	21–25 years (%)	51.6	52.0 *	45.4
	26–30 years (%)	30.1 ^†^	30.3	32.5 ^†^
	>30 years (%)	3.9 *	5.6 *	11.9 ^†^
WOMEN	14–20 years (%)	26.8	23.2	25.8
	21–25 years (%)	50.6	44.6	48.7
	26–30 years (%)	18.0	26.6 *^#^	20.0
	>30 years (%)	4.6	5.5	5.5

* Significant differences in comparison to the 10’s decade; ^#^ Significant differences in comparison to the 90’s decade (*p* < 0.01); ^†^ Significant differences in regards to women (*p* < 0.01).

**Table 2:** Distribution of the number of years remaining in the World Ranking top 100 badminton players.

Number of Years in the Top 100	Men (*n* = 593)	Women (*n* = 638)
1–3 years (%)	55.1	57.8
4–6 years (%)	21.6	18.7
7–9 years (%)	14.3	16.3
>9 years (%)	8.9	7.2

## Data Availability

Date available online: https://bwfbadminton.com/rankings/, accessed on 1 February 2021.
